# Enhancing sexual and reproductive health decision-making skills in underserved communities in Ghana: A quasi-experimental study

**DOI:** 10.1371/journal.pgph.0004733

**Published:** 2025-07-24

**Authors:** Jacqueline Nkrumah, Aaron Asibi Abuosi, Lily Yarney, Gordon Abekah-Nkrumah, Anita Asiwome Adzo Baku

**Affiliations:** 1 University of Education, Winnbena, Department of Health Administration and Education, Central Region, Ghana, West Africa; 2 University of Ghana Business School, Department of Health Services Management, Accra-Legon, Greater Accra Region, Ghana, West Africa; Health Policy Research Group, University of Nigeria, NIGERIA

## Abstract

Adolescent sexual and reproductive health (SRH) decision-making is crucial for long-term well-being. It plays a significant role in public health efforts, including reducing sexually transmitted infections (STIs) and teenage pregnancies. While cultural and socio-political influences on adolescent sexual and reproductive health (SRH) decision-making are well-documented, less is known about the effect of SRH educational materials’ difficulty levels on decision-making, particularly in Ghana and Sub-Saharan Africa. This study used a quasi-experimental design to assess the effectiveness of SRH educational materials of varying difficulty levels on decision-making skills using 317 adolescents, ages 11–15. Participants were assigned to a control group or one of the three intervention arms: difficult text, simplified text-only, or picture-enhanced text. Over six weeks, weekly sessions were conducted, followed by an end-line assessment with 249 participants and an 8-week post-intervention evaluation. Data analysis employed SPSS (version 26.0) and STATA (version 15.0), utilizing difference-in-difference and high-dimension fixed-effects models, paired sample t-test, and Kendall’s coefficient of concordance. Results show participants (66%) were females and were 7^th^ graders (56%). All three treatments, difficult text (10.808 points; CI = 1.2–20.36; P = 0.027), simplified text (11.60 points; CI = 4.68–18.52; P = 0.001), and picture-enhanced text (11.145 points; CI = 3.96–18.32; P = 0.002) significantly improved adolescents’ decision-making scores. After controlling for time-invariant characteristics within groups, the difficult-text material’s effect on decision-making scores declined (β3 = -6.442 points; CI = -13.908 to 1.096; P = 0.094) while that of the simplified and picture-enhanced materials was maintained. The study highlights the need for well-designed materials and effective pedagogy in SRH education to enhance learning and retention. Incorporating structured reading and discussion-based sessions into the school health program is recommended to promote the real-world application of SRH information and improve long-term SRH outcomes in adolescents.

## Introduction

Sexual and reproductive health (SRH) education and literacy are crucial for adolescents, particularly in Sub-Saharan Africa (SSA), where complex health, social, and developmental challenges persist. Effective educational interventions can significantly impact adolescent health outcomes by delaying sexual debut, promoting abstinence, enhancing knowledge of modern contraceptives, dispelling misconceptions about contraceptive effectiveness, and reducing high-risk sexual behavior [1–3]. The adolescent period is crucial for introducing SRH education, as it influences adolescents’ health, economic potential, and well-being [4]. SRH decision-making skills are linked to interactive health literacy [5], which involves an individual’s ability to read, interpret, and apply health information to make informed decisions. The capacity to make decisions is vital for adolescents and young people to cultivate safe and healthy sexual practices [6,7]. Well-informed adolescents have control over their bodies and choices that protect their future. Promoting SRH decision-making skills among adolescents will reduce health inequalities and improve gender equity [8]. Most importantly, adolescent decisions can significantly affect their long-term health and align well with the goal of public health, such as lowering sexually transmitted infections (STIs), teen pregnancy, and recurrent pregnancy [6,9].

Adolescence comes with major transitions, including the onset of puberty, sexual activities, consensual relationships, and childbearing. Vulnerability increases among young adolescents due to limited access to appropriate SRH education, and sexual coercion or violence [10]. Findings also point to lower SRH decision-making capacity among adolescents aged 15–19. Studies have emphasized that the lack of accurate information, as well as individual characteristics and social norms regarding qualifications for contraceptive use and side effects, stigma, and lack of privacy at the institutional level are factors affecting contraceptive use decision-making among adolescents in SSA [11–13]. Interventions to remove these barriers and promote adolescents’ SRH decision-making abilities are critical for reducing unfavorable outcomes such as teenage pregnancy and high-risk sexual behavior.

In Ghana, teen pregnancy continues to be a significant issue [14,15]. The country has a pooled prevalence of 15.4% from 1988-2019 [16]. The country continues to record an increasing number of pregnancies among young and older adolescents. Records between 2016–2020 show that 542,131 girls aged 15–19 and 13,444 girls aged 10–14 got pregnant in the country [17]. The literature on Ghana has also established low literacy as one of the factors related to teenage pregnancy [16,18]. Blunch [18] found that the ability to read in the Ghanaian and English languages was more strongly associated with reduced teenage pregnancy.

While studies in Ghana emphasize SRH education and literacy as essential in addressing adolescent SRH needs [16,19,20], existing policies primarily focus on SRH education, with limited attention to literacy. Policies such as the Adolescent Health Service Policy and Strategy, the National HIV and AIDS, STI Policy, and comprehensive sexuality education promote SRH education in schools [21–23]. Yet these policies face delivery challenges due to the widespread preference for abstinence-based approaches in Ghanaian society. An assessment of adolescents in Ghana reveals that few adolescents aged 10–14 can read at a grade-three level [24]. Given their poor reading skills, access to easy-to-understand SRH information is vital for improving their SRH literacy and outcomes [25,26]. Evaluation studies on SRH literacy interventions and their impact on adolescents’ decision-making abilities are essential to guide resource allocation toward effective approaches [27]. However, studies exploring how SRH literacy interventions can enhance adolescent decision-making are scarce in Ghana.

The health literacy literature has accentuated the effectiveness of simplified and picture-enhanced texts in improving recall, literacy, and informed decision-making among low-literate patients [28–30]. Although simplified and easy-to-understand educational materials have shown utility in improving health literacy and decision-making capacity, their usefulness in advancing SRH decision-making skills among adolescents has received little scholarly attention. This study assessed the causal impact of SRH educational materials, varying in difficulty level, on the decision-making skills of adolescents in basic schools located in underserved communities, using an SRH literacy intervention.

## Materials and methods

### Study area

Effutu Municipality, located in Ghana’s Central Region, was selected as the study area based on findings of studies in the Municipality indicating that over 60% of adolescents in basic schools engage in sexual relationships and 27% have low awareness of modern contraceptives [31]. Also, basic school adolescents have limited access to SRH information and education [20]. Anecdotal reports from the Municipal Health Directorate reveal a shortage of adolescent-friendly services, including Adolescent Health Corners, school-based SRH clubs, and educational materials. The Municipality has a population of 83,695, comprising 11,550 adolescents aged 10–14 and 8,872 aged 15–19. It consists of four sub-municipalities: Winneba East, Winneba West, Essuekyir-Gyahadze, and Kojo-Bedu North Low-Cost, with a total of 50 Junior High Schools (JHSs). Essuekyir-Gyahadze is predominantly rural, while Winneba East and Winneba West are characterized by slum areas inhabited largely by fisherfolk. Kojo-Bedu North Low-Cost features planned communities in the south and a mix of older and newer settlements in the north. The distribution of JHSs is as follows: Winneba East (6 public, 9 private), Winneba West (7 public, 1 private), Kojo-Bedu North Low-Cost (9 public, 8 private), and Essuekyir-Gyahadze (6 public, 4 private).

### Research design and study population

This prospective study employs a quasi-experimental design to assign participants to control and intervention groups based on location, minimizing cross-over and contamination risks. The aim was to assess the effectiveness of SRH learning materials, varying in difficulty level, in enhancing decision-making skills of in-school adolescents. The study population includes in-school adolescents in grades 7 and 8, aged 11–15, in Effutu Municipality. Although the initial target was adolescents aged 10–14, most students were between 12 and 19, necessitating an adjustment to 11–15 years. Grades 7 and 8 were included in the study to address the research gap created by increased focus on older adolescents [32]. This study is the third phase of a bigger study that restructured existing SRH educational materials to improve the SRH literacy of in-school adolescents in the Effutu Municipality.

### Eligibility criteria

Eligible participants were aged 11–15, specifically 7th or 8th graders residing in the Effutu Municipality. Participants needed to pass all three levels of screening (general literacy, visual acuity, and color perception), obtain parental consent, and provide written assent.

### Sample determination and size

**Fig 1:**
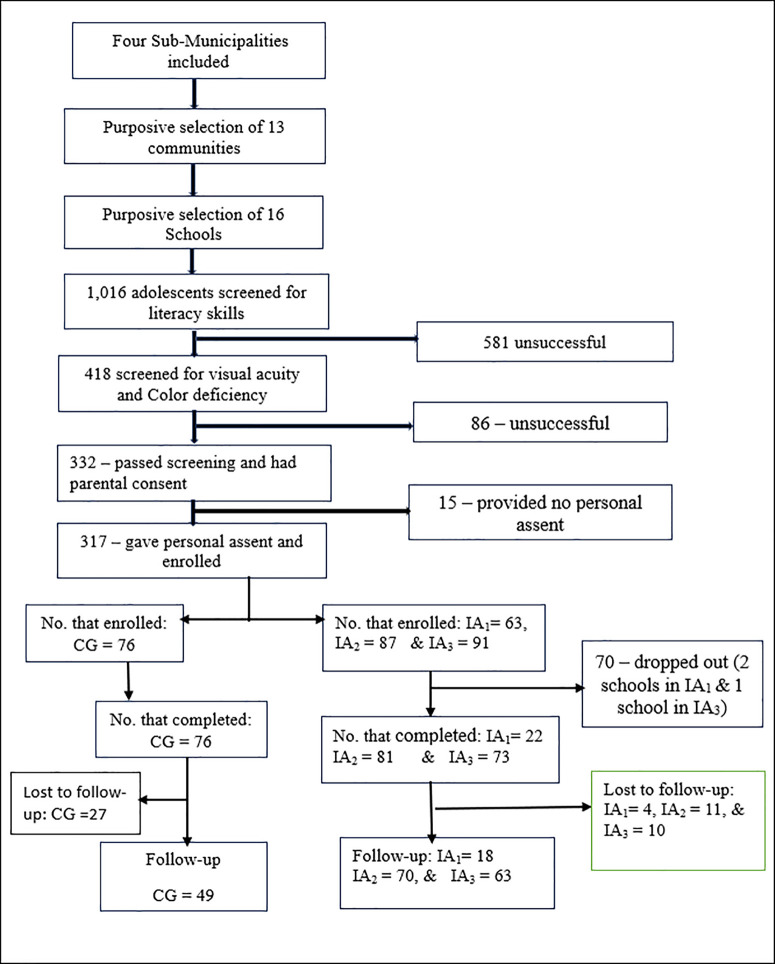
Participation Flowchart for the Intervention.


Fig 1 presents the flow chart process of the study sampling. We used a multistage sampling technique. All four sub-municipalities of the Effutu Municipality were included in the first stage. In the second stage, 13 communities were conveniently sampled based on the presence of a basic school, proximity to other communities with schools, and school management’s acceptance of the intervention. Communities with basic schools and shared boundaries were grouped into clusters. Five communities and schools were conveniently selected from Kojo-Bedu North Low-Cost. Winneba East and Winneba West each contributed two communities and three schools, while Essuekyir/Gyahadze had four communities and schools participating. A total of 408 basic school adolescents aged 11–15 were projected for inclusion in the intervention, with the sample size estimated using a formula for comparing two proportions in randomized controlled trials [33]. This approach ensured broad participation and minimized the risk of contamination between the control group (CG) and intervention arms (IAs). The sampling calculation was based on a previous study [34] following the formula below.

$$\text{n }={\left[(\text{Z}\alpha /2\;+\text{ Z}\beta \right)}^{2}\times \;\{({\text{p}}_{1}(1-{\text{p}}_{1})\;+\;({\text{p}}_{2}\;(1-{\text{p}}_{2})]/{\left({\text{p}}_{1}\;-\;{\text{p}}_{2}\right)}^{2}.\text{ Where}:$$

P_1 _= percentage increase in knowledge in the intervention group of a previous study of Dowse et al. [33] = 0.32.

P_2_ = percentage knowledge increases in the control group of Dowse et al.’s study [33] = 0.061.

Z_α/2_ = level of significance (5%) = 1.96 and Z_β_ = type two error or power (80%) = 0.84.

When plugged into the formula, the equation yielded n = 92 adolescents aged 11–15. The sample size was adjusted to include a 10% dropout rate. Using the formula, N1 = n/(1-d). N1 = 92/ (1 - 0.1) = 92/0.9 = 102. Therefore, N1 = 102 adolescents aged 11–15 years. On this basis, 102 participants were anticipated for selection into the CG and 102 for each IA: IA_**1**_, IA_**2**_, and IA_**3**_, making up the 408 expected participants.

To qualify as participants, basic school adolescents underwent a three-level screening process. First general literacy skills were assessed using a 526-word comprehension passage with 10 questions from the Progress in International Reading Literacy Study (PIRLS), those on reading for literacy experience [35], and five numeracy questions from the Trends in International Mathematics and Science Study (TIMSS), specifically, the 4th graders questions [36].

The assessment, conducted in participating schools with management’s permission, lasted 25 minutes. Scoring was based on the percentage of correct responses, graded using Ghana’s Basic Education Certificate Examination (BECE) system. Adolescents with literacy skill aggregates of 7–9 (below 50%) and 1–2 (70% or more) were considered to evaluate the contribution of literacy skills to SRH literacy. This analysis is the subject of a separate manuscript currently being prepared. At the end of the literacy skill assessment, 435 adolescents aged 11–15 out of the 1,016 qualified for the next level of screening. An ophthalmic nurse and trained assistants conducted visual acuity and color deficiency screening at the second and third levels. After these screenings, 418 adolescents qualified for the study, with 332 parental consents obtained. Ultimately, 317 adolescents agreed to participate and enrolled in the intervention. All screenings were conducted in schools during school hours between February and March 2022. (See S1 Table for details of schools that enrolled, completed, and were followed up).

### Intervention materials

A simplified and easy-to-understand SRH educational material [37] developed by the first author was used as the intervention material. The educational material was developed based on the SRH literacy needs of in-school adolescents in the Effutu Municipality [20,38]. SRH texts available online and in print were gleaned and synthesized into a single text and simplified into an easy-to-understand educational resource based on the literacy needs assessment results. The easy-to-understand resource was presented in two formats: simplified text only and picture-enhanced format [37]. We used the synthesized text (difficult-text material) as the comparison educational resource in the intervention. All three formats of educational materials (i.e., the difficult text, the simplified text-only, and the picture-enhanced) had six main lessons. They included: pubertal changes and menstruation, sexual activities, abstinence, friendship, sexually transmitted infections (STIs), and contraceptives and methods.

### Data collection tools

Two primary instruments were created for data collection in this study: the Test of Decision-Making Skills in SRH (TD-SRH) and the SRH literacy intervention feedback questionnaire. The TD-SRH is a self-developed tool that evaluates young adolescents’ ability to apply knowledge gained from reading and discussing the educational materials to make informed SRH decisions. The authors could not locate a suitable validated instrument to fulfill the needs of this study, as existing tools almost always assess SRH knowledge, functional literacy, and contraceptive use rather than decision-making skill [39]. The tool’s development was guided by Anderson & Krathwohl’s methods for assessing higher-order cognitive skills [40]. The approach has been used in the medical field to evaluate clinical decision-making skills. Common cognitive assessments in this area include the key feature problem (KFP) [41] and the Modified Essay Questions (MEQ), introduced by the Royal College of Medical Practitioners [42]. The MEQ involves short clinical scenarios with structured questions and scoring formats [43]. Participants answer specific questions based on scenarios to demonstrate their cognitive and decision-making skills. The Key Feature Problem (KFP) focuses on essential problem-solving steps, using a structured multiple-choice format that allows for multiple correct answers, an improvement over the traditional multiple-choice method [44]. It shares similarities with the Modified Essay Question (MEQ), as both assess cognitive abilities like knowledge recognition, reasoning, and problem-solving [45].

Drawing from these techniques, the TD-SRH tool was developed to evaluate young adolescents’ decision-making skills in SRH contexts. It presents a 348-word scenario involving SRH issues, such as puberty, menstruation, STIs, pregnancy, unsafe sex, and sources of SRH information. Based on this scenario, structured questions in multiple-choice formats were designed to elicit thoughtful responses. The TD-SRH includes 10 question items, each with eight alternative answers (A-H), requiring participants to select two or more correct answers. This design aims to capture realistic decision-making processes and minimize the chance of random guessing, ensuring that participants demonstrate their understanding of SRH issues. The TD-SRH had two forms: TD-SRH_0_ and TD-SRH_1_. TD-SRH_1_ modifies the first form by varying and rearranging question items. This process ensured reliability in testing participants’ decision-making skills effectively. The instrument was assessed for content validity. A health education and medical practitioner, and two experts in assessment and evaluation, validated the content of the questionnaire. Their feedback was used in revising the first and second forms. We further pre-tested the questionnaire among 75 basic school adolescents (35 for TD-SRH_0_ and 40 for TD-SRH_1_) recruited from schools excluded from the intervention. The test instruments were checked for reliability using Cronbach’s alpha.TD-SRH_0_ had an alpha value of 0.70, and TD-SRH_1_ had an alpha of 0.70. (See S1 File for the TD-SRH_0_ and TD-SRH_1_).

The intervention feedback questionnaire was divided into two parts. The first part had four items with Yes and No responses. It sought to ascertain whether participants could learn the materials at home and the support they received in learning the materials. The second part has 10 items on a 5-point Likert scale with responses ranging from strongly agree (1) to strongly disagree (5). Where strongly agree means participants concur with the statement, and strongly disagree means participants object to the statement. The last item has responses ranging from very much (1) to not at all (5), where very much means that participants liked the lessons, and not at all means they did not like the lessons learned. (See S1 File for the intervention feedback questionnaire.)

### Intervention and data collection

The intervention was organized on the premises of the selected schools. We followed three steps to roll out the intervention. The first step involved collecting baseline data on decision-making skills using the TD-SRH_0_ test, completed in 18–20 minutes. Data collection was done by the first authors and the trained research assistants. In step two, schools and their participants were randomly assigned by location to the CG or IA_1_-IA_3_. Schools in Winneba East were assigned to CG (no treatment), Winneba West to IA_1_ (consuming difficult-text material), Essuakyir-Gyahadze to IA_**2**_ (using the simplified text-only), and Kojo-Bedu North Low-Cost to IA_**3**_ (consuming the picture-enhanced material). Educational materials were distributed to the treatment groups and copies to school management one week before the intervention, allowing parents and school managers to know the contents that the adolescents would consume.

Step three involved weekly reading sessions, where participants read passages from the materials at home and took turns reading their assigned pages aloud to the hearing of other participants in the meeting to ensure they covered all lessons. Sessions were held separately for males and females but concurrently, with class sizes ranging from 4 to 20 participants. Each session lasted one hour, with 40 minutes for reading, on average, and 20 minutes for questions and answers. This process ensured active engagement and comprehension while addressing participants’ SRH literacy needs. A focal teacher in each school oversaw arrangements for the sessions.

The first author and ten research assistants (five males, five females), aged 21–25, moderated reading, questions, and answer sessions in participating schools. The assistants, third-year Health Administration and Education students from the University of Education, Winneba, were trained to use the SRH educational materials and facilitate discussions. They received the trainers’ version of the SRH educational materials to study and prepare for the lessons. This version contained more detailed information on each topic than the participants’ educational materials. The assistants’ age range aligned with the preferences of adolescents, as many of them feel reluctant to engage in adult-adolescent SRH conversations [20]. We concluded the intervention at the end of the sixth week. However, school heads scheduled the data collection during the one-hour weekly meeting, which allowed for end-line data collection in the seventh week using the TD-SRH_1_ test instrument. The moderators of the reading session gathered the data. However, we reassigned them at the end-line to minimize the familiarity effect and ensure consistency and objectivity. A maximum of 25 minutes was used for the end-line test. The intervention was conducted between May and July 2022. The decision-making test was readministered eight weeks after the end-line data collection. This follow-up assessment aimed to evaluate how well adolescents retained the knowledge and understanding gained from the lessons and their ability to apply this knowledge to SRH-related decision-making. The assessment was conducted using TD-SRH_o_ in the last week of September 2022.

### Data processing and analysis

The sociodemographic data were organized into two main categories: respondents’ demographic information and the description of decision-making scores. The demographic information included gender, age, grade level, place of residence, meeting attendance, and literacy skills. Attendance and grade level were recorded as numeric variables in their original form. Gender was coded as (0) for males and (1) for females, school type as (0) for private schools and (1) for public schools, and place of residence as (0) for rural and (1) for urban. Data from the intervention feedback questionnaire were recorded by maintaining the numeric values assigned to each response on the Likert scale to preserve its ordinal nature. Regarding the TD-SRH_0_ and TD-SRH_1_, each correct response was awarded one point, while incorrect answers received zero points. We calculated the percentage of correct answers by dividing the total number of correct responses by the total number of questions and multiplying the results by 100 to obtain a percentage score. Descriptively, SRH decision-making skills were categorized into three: adequate (75–100), moderate (60–74), and inadequate (0–59).

All data were entered into Microsoft Excel 2013 for preliminary organization. The dataset was subsequently processed and analyzed using two statistical software packages: SPSS (version 26.0) and STATA (version 15.0). STATA was used for the difference-in-difference (DiD) and the high-dimensional fixed-effects (HDFE) models, as our version of SPSS did not support this analysis. We checked for baseline equivalence based on a Kruskal-Wallis H test to assess differences in pre-intervention SRH decision-making skill scores across the four intervention groups (n = 249). The results indicated no statistically significant differences in baseline decision-making scores between groups, X^2^ (3) = 3.889, p = 0.274.

Participants’ backgrounds and other information were analyzed using descriptive statistics. We used a paired sample t-test to estimate the reduction or otherwise in SRH decision-making scores of participants in the IAs and the CG eight weeks post-intervention. Kendall’s coefficient of concordance and test statistics were performed to determine participants’ relative agreement with the statements in the feedback questionnaire.

SRH educational materials’ effect on participants’ decision-making skills was assessed using DiD to compare changes in decision-making skills between the IAs and the CG across time expressed as: Yi,t=a+β1Ti+β2Pt+β3TiPt+εi,t.

Where: Yi,t is the outcome variable, decision-making skills of adolescent *I,* T*i* (1 = participated, 0 = did not participate) represents participation in the intervention. Pt (0 = pre-intervention, 1 = post-intervention) means the period. Ti*Pt measures the interaction effect (1 when participation and post-intervention period apply). ԑ**i,t** is the error term, and parameters a, **β1, β2, β3** are defined as:

**a:** average outcome of non-participants pre-intervention.
**β1:** the initial difference between intervention and control groups.
**β2:** change in outcomes of non-participants over time
**β3:** effect of the SRH literacy intervention.

This model estimated the intervention’s impact by analyzing differences in decision-making skills between groups and periods.

### Statistical hypotheses for β3

The following statistical hypotheses were formulated:

**Ho1:** Difficult SRH educational materials do not significantly affect adolescents’ decision-making skills.
**Hi1:** Difficult SRH educational materials significantly affect adolescents’ decision-making skills.
**Ho2:** Simplified text-only SRH educational materials do not significantly affect adolescents’ decision-making skills.
**Hi2:** Simplified text-only SRH educational materials significantly affect adolescents’ decision-making skills.
**Ho3:** Picture-enhanced SRH educational materials do not significantly affect adolescents’ decision-making skills.
**Hi3:** Picture-enhanced SRH educational materials significantly affect adolescents’ decision-making skills.

We further performed HDFE linear regression to account for individual and group-level characteristics that do not vary with time and to provide a more conservative estimate of standard error and p-value. The model is expressed as: DMS=β0+β1treatment+β2post+β3(treatment x post+ fixed effect+ε. Where:

DMS is the outcome variable, SRH decision-making skills.
β0: the intercept, constant term, or average outcome when all variables equal zero.
β1: change in outcome associated with receiving the SRH educational intervention.
β2: the change in outcome associated with the post-treatment period.
β3: the change in outcome associated with receiving the treatment during the post-treatment period.
Fixed effect: is the unique ID provided for each intervention group and participants to account for unobserved heterogeneity across groups and to ensure a robust statistical inference.
ε:is the error term.

### Ethical considerations and ethics approval

We obtained written approval from the Municipal Education Service to conduct the study. We also obtained written permission and informed consent from participating adolescents’ school heads and parents. Each participating school head nominated a focal teacher to serve as a liaison between the school and the authors. Both the schools and parents were duly informed about the intervention, with opportunities provided for ongoing interaction with the first author. Furthermore, inclusion in the intervention was based on adolescents’ written assent. Ethics approval was obtained from the Ghana Health Service Ethics Review Committee in October 2021, ethics approval number GHS-ERC006/10/21.

## Findings

Table 1 presents background information and other descriptive data. The intervention recorded 317 participants, comprising 201 females and 116 males at baseline and 247 participants at end-line, giving a 78.5% completion rate over the number at baseline. Two hundred and one (201) participants were followed up 8 weeks after the end-line, representing an 81% follow-up rate over the number at the end-line. Most participants were 14 (34.9%) and 15 (37.8%) years old. Forty-five percent (45%) were rural dwellers, and 56% were 7^th^ graders. At baseline, 55% of participants had inadequate SRH literacy, which improved to moderate (42%) and adequate (17%) literacy at the end-line.

**Table 1:** Descriptive Information from the Intervention.

Background Information	Control and Intervention Arms Frequency				
	CG n (%)	IA_1_ n(%)	IA_2_ n(%)	IA_3_ n(%)	Total n(%)
***Baseline***	76	63	87	91	317
***End-line***	71	22	81	73	247
***8 post-intervention***	49	18	70	64	201
***Age***					
11	0	0	1 (1)		1 (0.4)
12	5 (7)	0	4 (5)	7 (9)	16 (6.4)
13	12 (17)	2 (9)	13 (16)	24 (32)	51 (20.5)
14	26 (37)	7 (32)	28 (35)	26 (34)	87 (34.9)
15	27 (39)	13 (59)	35 (43)	19 (25)	94 (37.8)
Mean	14 ± 0.92	14 ± 0.67	14 ± 0.94	13 ± 0.94	
***Gender***					
Male	27 (39)	7 (31.8)	26 (32)	25 (34.2)	85 (34)
Female	44 (61)	15 (68.2)	55 (68)	50 (65.8)	164 (66)
***School Type***					
Private	48 (69)	22 (100)	15 (19)	9 (11.8)	94 (38)
Public	22 (31)	0	66 (81)	67 (88.2)	155 (62)
***Place of Residence***					
Rural	17 (24)	2 (9.1)	64 (79)	29 (38.2)	112 (45)
Urban	53 (76)	20 (90.9)	17 (21)	47 (61.8)	137 (55)
***Grade Level***					
Basic 7	36 (51)	15 (68.2)	46 (57)	42 (55.3)	139 (56)
Basic 8	34 (49)	7 (31.8)	35 (43)	34 (44.7)	110 (44)
***Learning Ability***					
Lower ability	37(53)	15 (68)	40 (49)	37 (49)	129 (52)
Higher ability	33(47)	7 (32)	41 (51)	39 (51)	120 (48)
***Attendance***					
Median	–	6	5	6	
Minimum	–	2	2	2	
Maximum	–	6	6	6	
**Decision-making skills**					
***Baseline Scores***					
Adequate	15 (21)	0	9(11)	14 (19)	38 (15)
Moderate	22 (31)	5(23)	25(31)	22 (30)	74(30)
Inadequate	34 (48)	17(77)	47 (58)	37 (51)	135(55)
***Endline Scores***					
Adequate	4 (6)	0	15 (19)	23 (32)	42(17)
Moderate	30 (42)	7(32)	40 (49)	27(36)	104(42)
Inadequate	37 (52)	15(68)	26 (32)	23 (32)	101(41)

Table 2 presents the regression output that compares changes in SRH decision-making scores between the control group and the intervention arms IA_1_, IA_2_, and IA_3_. The adjusted R^2^ of the models explains 4.38%, 4.51%, and 5.57% of the variance in the decision-making abilities of participants in IA_**1**_, IA_**2**_, and IA_**3**_, respectively. The F statistics show that the T_**1**_ model explains 3.89%, P = 0.010, of the relationship between the intervention and the decision-making scores recorded among the participants. The relationship between the simplified text-only SRH materials and the decision-making scores of participants in IA_2_ was explained by 5.7% (5.70; P = 0.001) of the IA_**2**_ model. The IA_**3**_ model explains 6.59% (6.59; P = 0.056) of the relationship between the picture-enhanced materials and the decision-making scores of participants in IA_**3**_.

**Table 2:** DiD Estimates of Treatment Effect on Participants’ Decision-making Skills.

SRH Education Intervention Arms	Coefficient	Stand. Error	t-statistics	P-value	Adjusted r^2^	F	F Prob.	Confidence interval (95%)		
**Model 1 (IA**_**1**_)					0.0438	3.89	0.010			
Outcome at baseline (β_1_)	-11.632	3.42	3.40	0.001				-18.38	–	-4.87
Trends in SRH Literacy (β_2_)	-2.183	2.43	-0.90	0.371				-6.98	–	2.62
Intervention’s effect (β_3_)	10.808	4.84	2.23	0.027				1.25	–	20.36
Constant	58.507	1.72	33.99	0.001				55.11	–	61.90
**Model 2 (IA**_**2**_)					0.0451	5.70	0.001			
Outcome at baseline (β_1_)	-3.975	2.50	-1.59	0.113				-8.89	–	-0.95
Trends in SRH Literacy (β_2_)	-2.183	2.55	-0.86	0.393				-7.20	–	2.83
Intervention’s effect (β_3_)	11.601	3.52	3.30	0.001				4.68	–	18.52
Constant	58.507	1.80	32.43	0.001				54.95	–	62.05
**Model 3 (IA**_**3**_)					0.0557	6.59	0.056			
Outcome at baseline (β_1_)	-0.803	2.58	-0.31	0.756				-5.88		4.28
Trends in SRH Literacy (β_2_)	-2.183	2.58	-0.31	0.769				-5.88	–	4.28
Intervention’s effect (β_3_)	11.145	3.64	3.06	0.002				3.96	–	18.32
Constant	58.507	1.83	32.03	0.001				54.91	–	62.10

The β_1_ coefficients of the three models show that before the intervention, participants in the IA_1_, on average, had an 11.6-point (-11.632 points; CI = -18.38-4.87; P = 0.001) decrease in decision-making scores than those in the CG. In IA_2_, the participants had an average of 3.9 points (-3.975 points; CI = -8.89-0.95; P = 0.113) reduction in decision-making scores compared to the control group. In IA_3_ (-0.803 points; CI = -5.88 4.28 P = 0.756). The β_2_ coefficients (-2.183) indicate decision-making scores decreased by 2.18 points in the control group over the intervention period, suggesting that similar reduction would have been recorded in the three arms, IA_1_ (-2.183 points; CI = -6.98 –2.62; P = 0.371), IA_2_ (-2.183 points; CI = -7.20 –2.83; P = 0.393), and IA_3_ (-2.183 points; CI = -5.88–4.28; P = 0.769) without the intervention.

The β_**3**_ coefficients estimate the actual effect of the educational materials used for the intervention. The difficult-text material used by the IA_1_ participants positively and significantly enhanced participants’ decision-making scores (10.808 points; CI = 1.25– 20.36; P = 0.027) by 10.80 points. The simplified text-only material had a significant positive effect (11.60 points; CI = 4.68-18.52; P = 0.001) on decision-making scores by 11.60 points in the IA_2_. The picture-enhanced text material also had a significant positive effect (11.145 points; CI = 3.96-18.32; P = 0.002) on the decision-making scores of participants in the IA_3_ by 11.14 points compared to the CG.

The positive confidence intervals of the three models confirm that the intervention’s effect is positive and unlikely to occur by chance. Informed by the confidence intervals of the β3 of IA_**1**_, IA_**2**_, and IA_**3**_ models, the alternative hypotheses are accepted. We conclude that the three SRH educational materials positively affect basic school adolescents’ decision-making skills.

The results in Table 3 present the educational materials’ interactions with post-intervention status to influence adolescents’ decision-making skills. The adjusted R-squared (0.0951) indicates that the model explains 9.5% of the intervention, while the within R-squared shows that 7.2% of the variation is attributable to the intervention and treatment effects. The F-statistics (11.71; P = 0.0001) confirm a significant contribution from the intervention, post-intervention status, or their interaction in explaining SRH decision-making. The post-intervention interaction effect (β3) varied across groups. Among IA_1_ participants, who used difficult text materials, decision-making scores decreased by 6.44 points (β3 = -6.442 points; CI = -13.908 -1.096; P = 0.094), though the effect was marginal. IA_2_ participants, who received simplified text-only materials, showed a significant improvement (β3 = 12.171 points; CI = 5.626 - 18.715; P = 0.001) in decision-making score by 12.17 points.

**Table 3:** HDFE Estimates of Treatments’ Effect on Participants’ Decision-Making Skills.

SRH Education Intervention Arms	Coefficient	Robust Stand. Error	t-statistics	P-value	Adjus-ted r^2^	Within r^2^	F	F Prob.	Confidence interval (95%)		
					0.095	0.072	11.71	0.0001			
***Post period Effect β***_**2**_	-2.183	2.316	-0.94	0.347					-0.746	–	2.380
***Interaction Effects*: *β***_**3**_											
IA_**1**_	-6.442	3.827	-1.68	0.094					-13.980		1.096
IA _**2**_	12.171	3.322	3.66	0.001					5.626		18.715
IA _**3**_	11.772	3.143	3.75	0.001					5.580		17.963
***Constant***: ***β***_**0**_	56.349	1.027	54.87	0.001					54.326		58.372

IA_3_ participants, who used picture-enhanced materials, also experienced a statistically significant (β3 = 11.772 points; CI = 5.580 –17.963; P = 0.001) increase in decision-making score by 11.72 points. The confidence intervals for IA_2_ and IA_3_ confirm the robustness of these effects. The general post-intervention effect (β2 = -2.183 points; P = 0.347) indicates a slight decline in SRH decision-making scores after the intervention by 2.18 points, though the effect was not statistically significant, as reinforced by the confidence interval. This suggests that, overall, without the SRH literacy intervention, decision-making skills may not have improved. However, specific treatments, such as simplified text and picture-enhanced materials, proved effective in enhancing these skills. Since the estimation used a fixed-effect model, which captures within-group rather than between-group variation, participants in each group received the same treatment.

As revealed by the omitted coefficient of β1, within-group variation was absorbed by the fixed-effect model.

In Table 4, between the end-line and the 8-week follow-up, there were no statistically significant changes in SRH decision-making skills in any of the intervention arms, IA_**1**_ **=** -2.500 (t = -0.879; 95% CI = -7.644–4.311; P = 0.391), and IA_3 _= -1.328 (t = 0.494; 95% CI: -6.696–4.040; P = 0.623), or control group CG = -3.857 (t = -1.112; CI = -10.830–3.116; P = 0.272).

**Table 4:** Paired Sample Means of End-line and Eight weeks follow-up Scores for Participants’ SRH Decision-making Skills.

Paired Samples Statistics				Paired Sample Test							
Variables	Mean	Std. De-viation	Std. Error	Mean Difference	Std. Deviation	Std. Error	t-statistics	P-value	95% Conf. Interval		
	***No treatment - CG***										
***Endline***	55.51	15.281	2.183	-3.857	24.274	3.468	-1.112	0.272	-10.830	–	3.116
***8 weeks post-intervention***	59.37	16.058	2.294								
	***Difficult text material (IA***_***1***_ **)**										
***Endline***	56.11	8.512	2.006	-2.500	12.060	2.843	-0.879	0.391	-7.644	–	4.311
***8 weeks post-intervention***	58.61	10.982	2.588								
	***Simplified text-only material (IA***_***2***_ **)**										
**Endline**	65.36	11.831	1.424	4.551	21.577	2.598	1.752	0.084	-0.633	–	9.734
**8 weeks post-intervention**	60.81	18.462	2.223								
	***Pictograph-enhanced text material (IA***_***3***_ **)**										
***Endline***	67.89	13.538	1.692	-1.328	21.491	-2.696	0.494	0.623	-6.696	–	4.040
***8 weeks post-intervention***	69.22	16.034	2.117								

Table 5 provides descriptive statistics of participants’ feedback on the SRH educational sessions. The arithmetic mean values show that participants had a positive response to the question items on average. However, the mean ranks indicate the relative agreement for each question item in the feedback questionnaire based on how participants ranked them. Those ranked higher were: “I *could easily share my views with my schoolmates,”* (IA_1_ = 8.27), (IA_2 _= 7.65), and (IA_3 _= 6.99); “*The material was easy to understand”* (IA_1 _= 6.48), (IA_2 _= 6.47), and (IA_3 _= 6.61); and “*I was able to speak openly during the lessons*,” ((IA_1 _= 6.57), (IA_2 _= 6.28), and (IA_3 _= 6.27). The result suggests that the participants found the ability to share their views with peers, speak openly during the lessons, and understand the learning materials to be the most favorable aspects of the lessons.

**Table 5:** Participants’ Feedback on the SRH Literacy Intervention.

Description		IA_1_						IA_2_					IA_3_				
	Rank means	Mean	Ken. W	X^2^	P-value	Description	Rank means	Mean	Ken. W	X^2^	P-value	Description	Rank means	Mean	Ken. W	X^2^	P-value
I could easily share my views with my schoolmates.	8.27	2.59	0.14	30.83	0.001	I could easily share my views with my schoolmates.	7.65	1.80	0.094	77.48	0.001	I could easily share my views with my schoolmates.	6.99	1.66	0.067	49.52	0.001
I was able to speak openly during the lessons.	6.57	1.95				The material was easy to understand.	6.47	1.45				The material was easy to understand.	6.61	1.42			
The material was easy to understand.	6.48	1.82				I had time to share all my thoughts in all the lessons	6.28	1.46				I had time to share all my thoughts in all the lessons	6.27	1.41			
I had time to share all my thoughts in all the lessons	6.39	1.82				I was able to speak openly during the lessons.	6.15	1.43				I did not feel shy during the lessons.	6.07	1.34			
Overall, how did you like the lessons you have learned	6.20	1.77				I did not feel shy during the lessons.	6.03	1.43				The lessons were helpful to my daily life.	5.83	1.23			
The learning material was easy to read.	5.52	1.59				The learning material was easy to read.	5.75	1.29				I was able to speak openly during the lessons.	5.90	1.30			
The teacher answered all my questions.	5.39	1.73				Other JHS school pupils should be taught SRH lessons.	5.63	1.28				Other JHS school pupils should be taught SRH lessons.	5.63	1.26			
Other JHS school pupils should be taught SRH lessons.	5.18	1.50				The teacher answered all my questions.	5.45	1.28				The facilitator answered all my questions.	5.55	1.23			
The lessons were helpful to my daily life.	4.82	1.45				The lessons were helpful to my daily life.	5.37	1.24				The learning material was easy to read.	5.30	1.18			
I did not feel shy during the lessons.	4.70	1.41				Overall, how did you like the lessons you have learned	4.97	1.11				Overall, how did you like the lessons you have learned	5.28	1.18			

*IA*_*1:*_(Ken.W = 0.14, X2 = 30.83, p = 0.001*); IA*_*2:*_(Ken.W = 0.094, X2 = 77.48, p = 0.001*); IA*_*2:*_(Ken.W = 0.067, X2 = 49.52, p = 0.001*).*

The intervention feedback questionnaire was measured from Strongly Agree to Strongly Disagree. Where: 1 = strongly agree, 2 = agree,

3 = Not sure, 4 = Disagree, and 5 = Strongly disagree. The overall likeness for the lesson was measured as very much to not at all.

Where: 1 = Very much, 2 = Much, 3 = Not sure, 4 = Not much, and 5 = Not at all.

Lower ranked items were: “Overall, *how did you like the lessons you have learned”* (IA_2_ = 4.97), and (IA_3 _= 5.28); and “*I did not feel shy during the lessons”* (IA_1 _= 4.78) shows that there are areas of the intervention that may require attention to improve participants’ overall satisfaction of the intervention. Kendall’s W (IA_1 _= 0.14), (IA_2 _= 0.094), and (IA_3 _= 0.067) confirm participants had varied perceptions about the intervention’s effectiveness across different elements. The chi-square values (IA_1 _= 30.83; P = 0.001), (IA_2 _= 77.48; P = 0.001), and (IA_3 _= 49.52; P = 0.001) indicate that there is a statistically significant weak agreement regarding their perceived effectiveness of the intervention. They further suggest that the feedback is not due to random selection and that some aspects of the lessons were preferred over others.

## Discussion

This study assessed the impact of SRH educational materials of varying difficulty on adolescents’ decision-making skills. findings suggest that while general SRH education may be ineffective, structured and simplified materials improve informed decision-making. Despite ethical approvals, the intervention faced opposition from some school heads, who viewed SRH education as promoting sexual activity. This resistance underscores the persistent stigmatization of adolescents’ SRH in Ghana, consistent with prior research. Asampong et al. [46] found that parent-adolescent communication about sexuality in parts of southeastern Ghana is often tense, driven by fears of encouraging sexual activity or sparking curiosity [47]. These societal misconceptions reflect broader challenges in addressing adolescent SRH. In this study, some teachers opposed the picture-enhanced SRH materials, deeming them too explicit and potentially encouraging early sexual activity. Such reactions contributed to resistance and dropout. Future interventions may benefit from culturally sensitive materials, stakeholder engagement, and alternative delivery approaches to improve acceptance, enhance participant retention, and address deeply rooted stigmas around adolescent SRH.

Although initial descriptive comparisons suggested potential baseline differences, the Kruskal-Wallis H test indicated no statistically significant differences in the SRH decision-making skills across the group before the intervention. This supports the assumption of baseline equivalence and strengthens the internal validity of the design. The DiD analysis showed changes in SRH decision-making skills over time between the control group and the three IAs. At baseline, all IAs had lower decision-making scores. The negative β2 values suggest that, without the intervention, decision-making skills would have declined over time. Yet, the values are statistically not significant. However, the β_3_ coefficients show that the intervention significantly improved skills across all IAs, indicating its effectiveness. Each format of educational material positively impacted SRH decision-making compared to the control group, highlighting the potential of structured SRH literacy interventions to enhance adolescents’ decision-making abilities and counter potential skills deterioration over time. IA_1_ (difficult text) showed a marginal decline in decision-making under the fixed effect model,

contrasting with its DiD improvement, possibly due to the reduced sample size. This IA may have been underpowered to detect a similar but potentially meaningful effect. It is also possible that the DiD may have overestimated its effect due to baseline differences. Despite the reduced sample size, statistically significant positive effects were observed for IA_2_ and IA_3_, indicating a consistent effect large enough to be detected even under limited power. These findings lend credibility to the effectiveness of these interventions, emphasizing the need to align SRH educational materials with adolescents’ comprehension levels to improve decision-making and ensure intervention effectiveness.

Existing literature from low-middle-income countries (LMICs) highlights the effectiveness of school-based SRH education and planned behavior-based interventions in significantly enhancing knowledge, awareness, attitudes, and the uptake of SRH services [48,49]. In Iran, for instance, planned behavior-based SRH interventions have improved adolescents’ attitudes, subjective norms, perceived behavioral control, and intentions [50]. The findings also suggest that Ghana’s school health program and other co-curricular activities may be ineffective in enhancing adolescents’ ability to make basic SRH-related decisions. It also highlights the potential for a continued decline in adolescents’ SRH decision-making skills without targeted interventions, emphasizing the critical need for focused efforts in this area.

Decision-making ability is closely linked to interactive literacy [5] and is often considered an outcome of health literacy [51]. It has also been identified as a mediator between health literacy and health outcomes [52]. While previous studies have explored formats for presenting health educational materials and their effect on functional literacy, knowledge, self-efficacy, and recall, there is limited evidence of their impact on health-related decision-making among adolescents [53–55]. Nevertheless, a study on health animations revealed that simplified text-only and simplified text combined with pictures can improve informed decision-making among low-literate populations [28].

The present study demonstrates that school health and comprehensive sexuality education programs aiming to improve adolescents’ decision-making skills can succeed using simplified educational materials, since they can improve reading and comprehension. This study contributes to filling gaps in the health literacy literature by providing valuable evidence on how the presentation format of SRH educational materials can influence adolescents’ ability to make SRH-related decisions. Yet, further research is needed to reinforce and expand upon these findings.

The lack of significant changes between the end-line and the 8-week follow-up suggests that no delayed effect emerged during the follow-up period. It also indicates the need for ongoing engagement or longer-duration interventions to reinforce decision-making skills among adolescents. The existing literature appears to contain few studies examining the effectiveness of different formats of educational material on decision-making following interventions, which makes it difficult to contextualize these findings. Nevertheless, SRH interventions often show improvements in outcomes at 3–6-month follow-ups. For example, a randomized controlled trial in Nigeria reported gains in reducing risky sexual behavior at 3 months [56]. A Tanzanian study employing problem-based pedagogy recorded significant retention of soft skills for safe sexual behavior at both 3 and 6-month follow-ups. Factors such as gender, school location, and cognitive engagement are also associated with better post-intervention SRH knowledge retention [56,57].

Participants responded positively to the SRH educational sessions, demonstrating increased confidence in discussing SRH topics with peers, understanding the content, and expressing themselves. Their consensus on aspects such as effectiveness, engagement, trust, and comprehension suggest the intervention was well-received, regardless of the material format. However, areas for improvement included lesson delivery, program appeal, and material readability. The perceptions of basic school adolescents align with existing literature. Reviews of European adolescents’ satisfaction with SRH interventions indicate high acceptance and satisfaction [58]. Similarly, an SRH educational intervention for adolescents with cystic fibrosis highlighted the importance of integrating an SRH education program [59]. These findings reinforce the significance of providing accessible and engaging SRH education tailored to adolescents’ needs. Addressing delivery methods and material clarity can enhance intervention impact, ensuring better comprehension, engagement, and long-term benefits for adolescents’ SRH literacy.

## Limitations of the study

The study experienced a 21% dropout from baseline to end-line and an additional 19% dropout eight weeks post-intervention. The moderate enrollment was influenced by ethical constraints, cultural beliefs, and stigma surrounding adolescent SRH. As a result, the statistical power of the study to detect small to moderate effects was likely reduced, increasing the risk of type II error. It also suggests that some true effects may not have reached statistical significance, especially in IA_1_. These challenges highlight the need to balance ethical considerations with equitable access. Stakeholder engagement and parent/community involvement may help overcome these barriers and create a supportive environment. Future studies should employ larger sample sizes to validate findings, especially in the IAs, where the negative effect was underpowered for firm conclusions.

## Conclusion

This study employed a quasi-experimental design to assess the impact of SRH educational materials of varying formats and difficulty levels on the decision-making skills of basic school adolescents. The findings revealed that simplified text and picture-enhanced materials had a sustained effect even after controlling for within-group time-invariant characteristics, highlighting the importance of aligning SRH content with adolescents’ literacy levels. Follow-up assessments reinforced the role of these materials in enhancing recall and retention. Adolescents expressed high satisfaction and acceptance of the intervention, with certain aspects preferred over others, underscoring their strong interest in SRH education. The results further emphasized the need to integrate structured reading and discussion-based SRH sessions into school curricula to address gaps in Ghana’s school health program. Such an approach can empower adolescents, strengthening their decision-making agency and retention of knowledge. Also, revising Ghana’s adolescent SRH policies to prioritize accessible, user-friendly educational resources may encourage independent learning and informed decision-making.

## Supporting information

S1 Data Data from the SRH literacy intervention. (XLSX)

S1 File Questionnaires. (DOCX)

S1 Table Additional information on sampling. (DOCX)
