# Supplementary material for: Enhancing sexual and reproductive health decision-making skills in underserved communities in Ghana: A quasi-experimental study
Source: PLOS Glob Public Health. 2025 Jul 24;5(7):e0004733. doi: 10.1371/journal.pgph.0004733 (PMC12288995; doi:10.1371/journal.pgph.0004733)
Supplement: S1 File — (DOCX) [file pgph.0004733.s002.docx]

**Decision-making Skills in Sexual and Reproductive Health (DSSRH)**

**Form I**

**Index Number:………………………………..** Grade Level: ……………. Age:……………….

Gender: Male [ ]. Female [ ]. Region: Christianity [ ]. Islam [ ]. Others [ ].

Place of Residence: (write the name of your community) …………………………………………

**Instruction**

This test presents a scenario and ten multiple-choice questions with options **A** to **H**. Each question has more than one answer. You must **Read the Scenario** **below** and answer the following questions by circling the correct alphabet. This test is not part of your schoolwork, and your answers will not be graded. Your score would be kept secret from your schoolmates. Your score will be used purely for research purposes.

**Scenario**

Esi is a 14-year-old teenager with problems similar to those of Atto, Abeku, and Mansa. She is in the second year of Junior High School (JHS two). Like all other young teens, she has started having menses but feels it is too early for her to start menses. Esi has a 28-day cycle. Some days after her last menses, which began on 25^th^ May 2020, Esi started to itch in the vagina. She found some whitish fluid coming from her vagina. Also, her vagina became reddish. Esi thinks she has a yeast (white) infection.

Mansa is the same age as Esi. She is in JHS two. Even though she has started menses, she has little knowledge of STIs and childbearing matters. Mansa is in the same class as Abeku, her best friend. Mansa and Abeku attended a party organized for the final year students of her school on 6^th^ June 2021. Whiles at the party, the two friends went outside and sat in a quiet place in the dark, where no one could see them. They began touching each other's private parts and kissed each other. The two friends fell into sexual temptation and had sex. They both went back to the party venue sometime later. Mansa and Abeku left for their homes after the party ended around 8:00 pm. Their school went on vacation on 10^th^ June 2021. Mansa waited for her menses between 20^th^ June and 27^th^ July 2021, when the school had gone on vacation. Mansa got worried about delays in her menses. She wanted to know why she had not had her menses from 20^th^ June to 27^th^ July 2021.

Like most young teens, Atto is 13 years old and not yet mature. He has very little knowledge of physical changes at puberty and childbearing issues. Atto has been having wet dreams and early morning erection. He worries about his body's outward changes but is unwilling to talk to his caregivers. He has many questions on his mind and is looking for someone to help him understand why those changes are happening in his body.

Turn to the next page and answer the questions. Circle the correct answer's alphabet.

1. Is it fit for Mansa and Abeku to sit where no one could see them?

(Select up to 3 answers)

1. No, it is not fit because there is very little time for the party.
2. No, it is not fit because Abeku and Mansa are the opposite sex and can get attracted.
3. Yes, it is fit because Abeku and Mansa are from the same school.
4. Yes, it is fit because the two are friends.
5. No, it is not fit because they can be tempted to have sex.
6. No, it is not fit because the party could have ended without them.
7. No, it is not fit. If Abeku has bad manners, he can rape Mansa
8. No, it is not fit because they had to be in the party venue.
9. If Mansa is pregnant, what could the two friends have done to avoid the pregnancy? (Select up to 3 answers).
10. The two could have avoided sitting together in the dark place alone.
11. Mansa could have washed her vagina with urine after having sex.
12. Mansa could have agreed to have sex two days before ovulation.
13. Abeku and Mansa could have held each other's hands without touching their private parts.
14. They could have used the vagina sex method to prevent pregnancy.
15. Mansa could have taken the emergency contraceptive (EC) pill 72 hours before having unsafe sex.
16. They could have used a condom to prevent pregnancy.
17. Mansa could have told her parent about it.
18. If Esi had a yeast (white) infection, how could she have had it? (Select up to 3 answers).
19. Esi could have had the infection naturally.
20. Esi's infection could be the cause of gonorrhea (gono).
21. Esi should tell her parent/caregivers about the signs she has.
22. Her infection could be the cause of a stomach problem.
23. Esi did not wash her vagina with a ginger solution.
24. It is also possible that Esi does not keep her panties clean
25. She could be having waist problems.
26. She could also have a yeast infection from a dirty toilet she used.
27. How can Esi protect herself from having STIs in the future? Esi should ………… (Select up to 3 answers).
28. Say no to all forms of sexual activities.
29. Have oral sex only.
30. Have anal sex only.
31. If Esi cannot avoid having sex, she should use a condom whenever she has sex.
32. Wash her vagina with urine after having sex.
33. Esi should not have more than one boyfriend.
34. Use the withdrawal method anytime she has sex with a boy/man.
35. Use emergency contraceptives after having sex.
36. Apart from menses, what other signs of puberty should ESi be having? (Select up to 4 answers).
37. Esi's breast should begin to grow.
38. She should have delayed ovulation.
39. Her voice should remain unchanged.
40. Esi should grow hairs in her armpit and pubic areas.
41. Esi's hips should be getting broader.
42. She should have dry skin and wrinkles.
43. She should have a whitish eye.
44. Esi should start having oily skin.
45. Why has Mansa not had her menses? (Select up to 3 answers).
46. Mansa may be pregnant.
47. She could be having a small lining in her womb.
48. Mansa could be having pains in the lower part of her stomach.
49. Mansa could be eating poorly.
50. She has no illness at all.
51. Mansa could be sleeping too often.
52. She could be doing lots of exercises.
53. It is usual for girls to miss their menses.
54. Apart from wet dreams, what other outward signs of puberty would Atto have? (Select up to 3 answers).
    1. Atto would have muscles in the legs and arms.
    2. He would have bigger lips and eyes.
    3. Atto would also have broad shoulders and chest.
    4. Atto may develop long legs and hands.
    5. He may have a body odor.
    6. Atto would have keloids on his face.
    7. Atto can also develop big breasts and body shape.
    8. He would have big eyes and a mouth.
55. If Atto wants to understand more about puberty, which of the following people can better educate him? (Select up to 3 answers).
56. Atto's best friends.
57. The school girlchild educator.
58. Website operators.
59. School mates.
60. The school counselor.
61. A family planning nurse/doctor.
62. Community members.
63. His brothers and sisters.
64. Why is it not too early for Esi to start having menses? It is not early because……… (select up to 2 answers).
65. Menses can begin as early as eight years.
66. Esi is tall and fat.
67. By age 13, few girls would have had their first menses.
68. Esi is a spoilt child.
69. Esi has excess blood.
70. Esi is 14 years old and has passed the age of first menses.
71. Esi developed teeth early when she was a child.
72. It does not look like Esi is 14 years old.
73. What could Mansa and Abeku have gained if they had said no to sex? (Select up to 4 answers).
74. They could finish school and that too on time.
75. They could have spent a long time together.
76. Mansa and Abeku could have avoided pregnancy and STIs.
77. The two friends could have become worried over a long period.
78. They could have waited to find the right guy/girl.
79. Abeku and Mansa could have used pregnancy prevention injections.
80. They could have become best of friends.
81. They could have had time to learn a trade.

**Decision-making Skills in Sexual and Reproductive Health (DSSRH)**

**Form II**

**Index Number:………………………………..**

**Instruction**

This test presents a scenario and ten multiple-choice questions with options **A** to **H**. Each question has more than one answer. You must **read the scenario** **below** and answer the following questions by circling the correct alphabet. This test is not part of your schoolwork, and your answers will not be graded. Your score would be kept secret from your schoolmates. Your score will be used purely for research purposes.

**Scenario**

Esi is a 14-year-old teenager with similar problems as Atto, Abeku, and Mansa. She is in the second year of Junior High School (JHS two). Like all other young teens, she has started having menses but feels it is too early for her to start menses. Esi has a 28-day cycle. Some days after her last menses, which began on 25^th^ May 2020, Esi started to itch in the vagina. She found some whitish fluid coming from her vagina. Also, her vagina became reddish. Esi thinks she has a yeast (white) infection.

Mansa is the same age as Esi. She is in JHS two. Even though she has started menses, she has little knowledge of STIs and childbearing matters. Mansa is in the same class as Abeku, her best friend. Mansa and Abeku attended a party organized for the final year students of her school on 6^th^ June 2021. Whiles at the party, the two friends went outside and sat in a quiet place in the dark, where no one could see them. They began touching each other's private parts and kissed each other. The two friends fell into sexual temptation and had sex. They both went back to the party venue sometime later. Mansa and Abeku left for their homes after the party ended around 8:00 pm. Their school went on vacation on 10^th^ June 2021. Mansa waited for her menses between 20^th^ June and 27^th^ July 2021, when the school had gone on vacation. Mansa got worried about delays in her menses. She wanted to know why she had not had her menses from 20^th^ June to 27^th^ July 2021.

Like most young teens, Atto is 13 years old and not yet mature. He has very little knowledge of physical changes at puberty and childbearing issues. Atto has been having wet dreams and early morning erection. He worries about his body's outward changes but is unwilling to talk to his caregivers. He has many questions on his mind and is looking for someone to help him understand why those changes are happening in his body.

**Turn to the next page** and answer the questions that **follow by circling the correct answer's alphabet.**

- - - 1. Is it fit for Mansa and Abeku to sit where no one could see them?

(Select up to 3 answers)

1. No, it is not fit because there is very little time for the party.
2. No, it is not fit because Abeku and Mansa are the opposite sex and can get attracted.
3. Yes, it is fit because Abeku and Mansa are from the same school.
4. Yes, it is fit because the two are friends.
5. No, it is not fit because they can be tempted to have sex.
6. No, it is not fit because the party could have ended without them.
7. No, it is not fit. If Abeku has bad manners, he can rape Mansa
8. No, it is not fit because they had to be in the party venue.
   - - 1. If Mansa is pregnant, what could the two friends have done to avoid the pregnancy? (Select up to 3 answers).
9. The two could have avoided sitting together in the dark place alone.
10. Mansa could have washed her vagina with urine after having sex.
11. Mansa could have agreed to have sex two days before ovulation.
12. Abeku and Mansa could have held each other's hands without touching their private parts.
13. They could have used the vagina sex method to prevent pregnancy.
14. Mansa could have taken the emergency contraceptive (EC) pill 72 hours before having unsafe sex.
15. They could have used a condom to prevent pregnancy.
16. Mansa could have told her parent about it.
    - - 1. If Esi had a yeast (white) infection, how could she have had it? (Select up to 3 answers).
17. Esi could have had the infection naturally.
18. Esi's infection could be the cause of gonorrhea (gono).
19. Esi should tell her parent/caregivers about the signs she has.
20. Her infection could be the cause of a stomach problem.
21. Esi did not wash her vagina with a ginger solution.
22. It is also possible that Esi does not keep her panties clean
23. She could be having waist problems.
24. She could also have a yeast infection from a dirty toilet she used.
    - - 1. How can Esi protect herself from having STIs in the future? Esi should ………… (Select up to 3 answers).
25. Say no to all forms of sexual activities.
26. Have oral sex only.
27. Have anal sex only.
28. If Esi cannot avoid having sex, she should use a condom whenever she has sex.
29. Wash her vagina with urine after having sex.
30. Esi should not have more than one boyfriend.
31. Use the withdrawal method anytime she has sex with a boy/man.
32. Use emergency contraceptives after having sex.

5. Apart from menses, what other signs of puberty should ESi be having? (Select up to 4 answers).

1. Esi's breast should begin to grow.
2. She should have delayed ovulation.
3. Her voice should remain unchanged.
4. Esi should grow hairs in her armpit and pubic areas.
5. Esi's hips should be getting broader.
6. She should have dry skin and wrinkles.
7. She should have a whitish eye.
8. Esi should start having oily skin.
9. Why has Mansa not had her menses? (Select up to 3 answers).
10. Mansa may be pregnant.
11. She could be having a small lining in her womb.
12. Mansa could be having pains in the lower part of her stomach.
13. Mansa could be eating poorly.
14. She has no illness at all.
15. Mansa could be sleeping too often.
16. She could be doing lots of exercises.
17. It is usual for girls to miss their menses
18. Apart from wet dreams, what other outward signs of puberty would Atto have? (Select up to 3 answers).
19. Atto would have muscles in the legs and arms.
20. He would have bigger lips and eyes.
21. Atto would also have broad shoulders and chest.
22. Atto may develop long legs and hands.
23. He may have a body odor.
24. Atto would have keloids on his face.
25. Atto can also develop big breasts and body shape.
26. He would have big eyes and a mouth.
27. If Atto wants to understand more about puberty, which of the following people can better educate him? (Select up to 3 answers).
28. Atto's best friends.
29. The school girlchild educator.
30. Website operators.
31. School mates.
32. The school counselor.
33. A family planning nurse/doctor.
34. Community members.
35. His brothers and sisters.
36. Why is it not too early for Esi to start having menses? It is not early because……… (select up to 2 answers).
37. Menses can begin as early as eight years.
38. Esi is tall and fat.
39. By age 13, few girls would have had their first menses.
40. Esi is a spoilt child.
41. Esi has excess blood.
42. Esi is 14 years old and has passed the age of first menses.
43. Esi developed teeth early when she was a child.
44. It does not look like Esi is 14 years old.
45. What could Mansa and Abeku have gained if they had said no to sex? (Select up to 4 answers).
46. They could finish school and that too on time.
47. They could have spent a long time together.
48. Mansa and Abeku could have avoided pregnancy and STIs.
49. The two friends could have become worried over a long period.
50. They could have waited to find the right guy/girl.
51. Abeku and Mansa could have used pregnancy prevention injections.
52. They could have become best of friends.
53. They could have had time to learn a trade.

**Intervention Feedback Questionnaire**

Participant I/D: ……………………

1. Were you able to read all the lessons in the house? Yes [ ]. No [ ].
2. Which of the following were you able to do? I was unable to learn the SRH after school [ ].

I learned SRH lessons with friends after school [ ]. I learned SRH lesions with my parents' support after school. [ ].

1. Did you take part in all the reading and question sessions? Yes [ ]. No [ ].

***Views on the Lessons***

For the list of questions in the table, please, tick (√) as suitable.

| **Perception Lessons Delivery** | Strongly agreed | Agreed | Not sure | disagree | Strongly disagree |
| --- | --- | --- | --- | --- | --- |
| The learning material was easy to read. |  |  |  |  |  |
| The material was easy to understand. |  |  |  |  |  |
| All lessons taught followed the time allowed. |  |  |  |  |  |
| I was able to speak openly during the lessons. |  |  |  |  |  |
| I had time to share my thoughts in all the lessons. |  |  |  |  |  |
| The teacher answered all my questions. |  |  |  |  |  |
| I could easily share my views with my schoolmates. |  |  |  |  |  |
| I did not feel shy during the lessons. |  |  |  |  |  |
| The lessons were helpful to my daily life. |  |  |  |  |  |
| Other JHS school pupils should be taught SRH lessons. |  |  |  |  |  |
|  | | | | | |
|  | Very much | Much | Not sure | Not much | Not At all |
| Overall, how did you like the lessons you have learned |  |  |  |  |  |
