# Supplementary material for: Enhancing sexual and reproductive health decision-making skills in underserved communities in Ghana: A quasi-experimental study
Source: PLOS Glob Public Health. 2025 Jul 24;5(7):e0004733. doi: 10.1371/journal.pgph.0004733 (PMC12288995; doi:10.1371/journal.pgph.0004733)
Supplement: S1 Table — (DOCX) [file pgph.0004733.s003.docx]

**S1 Table: Additional Information on Schools and Adolescents Included in the Study**

| **Baseline** | | | | | | | |
| --- | --- | --- | --- | --- | --- | --- | --- |
| **Control Group** | | **Intervention Arm 1** | | **Intervention Arm 2** | | **Intervention Arm 3** | |
| ***JHS*** | ***No. of part.*** | ***JHS*** | ***No. of part.*** | ***JHS*** | ***No. of part.*** | ***JHS*** | ***No. of part.*** |
| Don Bosco A | 20 | Unipra inclusive | 27 | Ateitu | 20 | Municipal Assembly | 16 |
| Don Bosco B | 35 | Cynclare | 12 | Ampah Prep. | 22 | Ntakorfam | 25 |
| Odobiriba | 21 | Zion B | 24 | Attekyedo | 8 | Ansaful | 22 |
|  |  |  |  | Gyahadzi | 19 | Blessed Assurance | 6 |
|  |  |  |  | Gyengyanadzi | 18 | New Winneba | 22 |
| **Total** | **76** |  | **63** |  | **87** |  | **91** |
|  | | | | | | | |
| **End-line** | | | | | | | |
| Don Bosco A | 19 | Unipra inclusive | 0 | Ateitu | 20 | Municipal Assembly | 0 |
| Don Bosco B | 32 | Cynclare | 0 | Ampah Prep. | 20 | Ntakorfam | 25 |
| Odobiriba | 21 | Zion B | 22 | Attekyedo | 7 | Ansaful | 22 |
|  |  |  |  | Gyahadzi | 17 | Blessed Assurance | 5 |
|  |  |  |  | Gyengyanadzi | 17 | New Winneba | 21 |
| **Total** | **72** |  | **22** |  | **81** |  | **73** |
|  | | | | | | | |
| **8 weeks follow-up** | | | | | | | |
| Don Bosco A | 19 | Unipra inclusive | 0 | Ateitu | 19 | Municipal Assembly |  |
| Don Bosco B | 30 | Cynclare | 0 | Ampah Prep. | 15 | Ntakorfam | 21 |
| Odobiriba | 0 | Zion B | 18 | Attekyedo | 7 | Ansaful | 22 |
|  |  |  |  | Gyahadzi | 14 | Blessed Assurance | 5 |
|  |  |  |  | Gyengyanadzi | 15 | New Winneba | 16 |
| **Total** | **49** |  | **18** |  | **70** |  | 64 |
